# Supplementary figures and images for: Whole-Transcriptome Profiling and Functional Prediction of Long Non-Coding RNAs Associated with Cold Tolerance in Japonica Rice Varieties
Source: Int J Mol Sci. 2024 Feb 15;25(4):2310. doi: 10.3390/ijms25042310 (PMC10889138; doi:10.3390/ijms25042310)

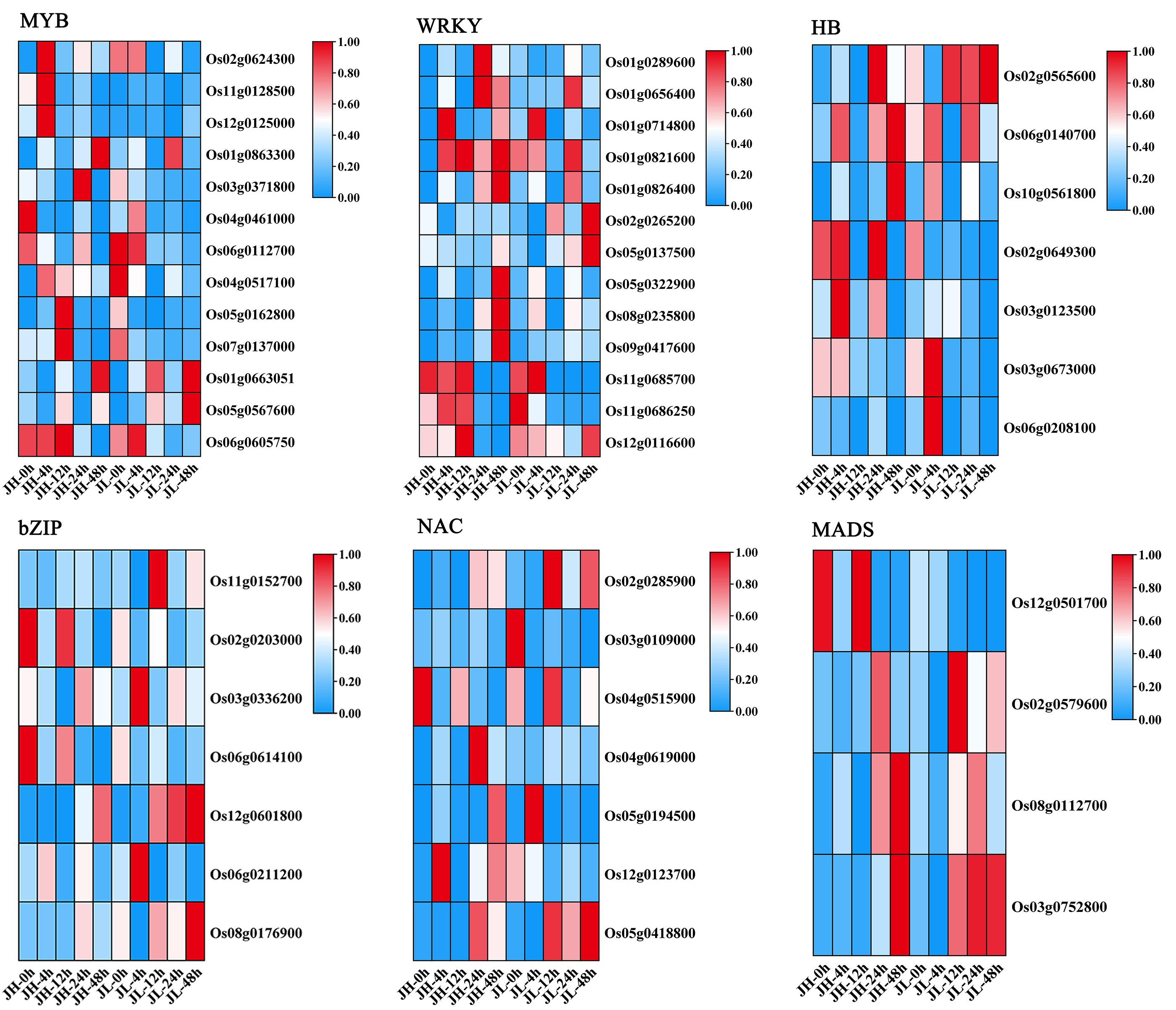

Supplement: Supplementary file 1 [file ijms-25-02310-s001.zip › Supplementary Figure S1.tif]

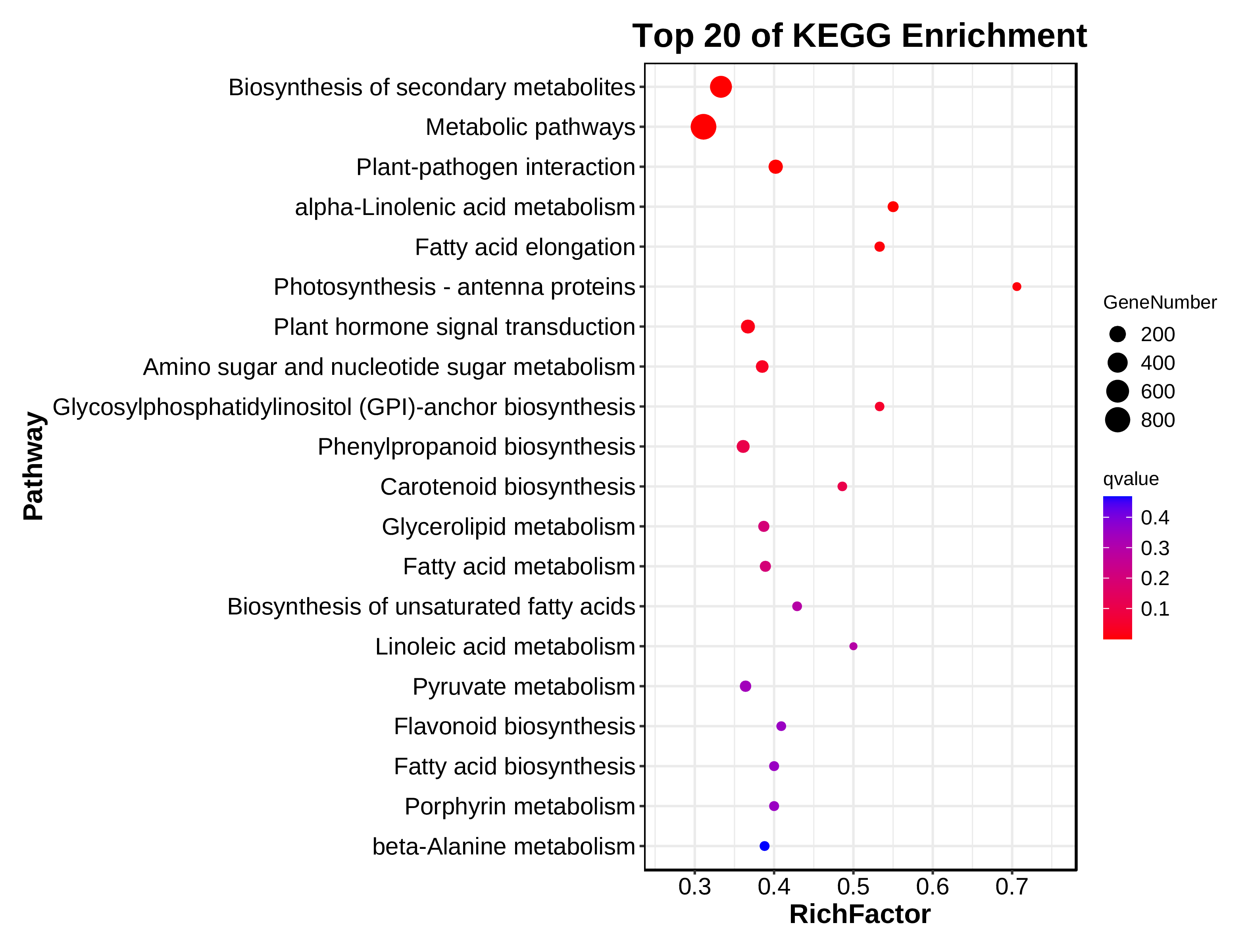

Supplement: Supplementary file 1 [file ijms-25-02310-s001.zip › Supplementary Figure S2.png]

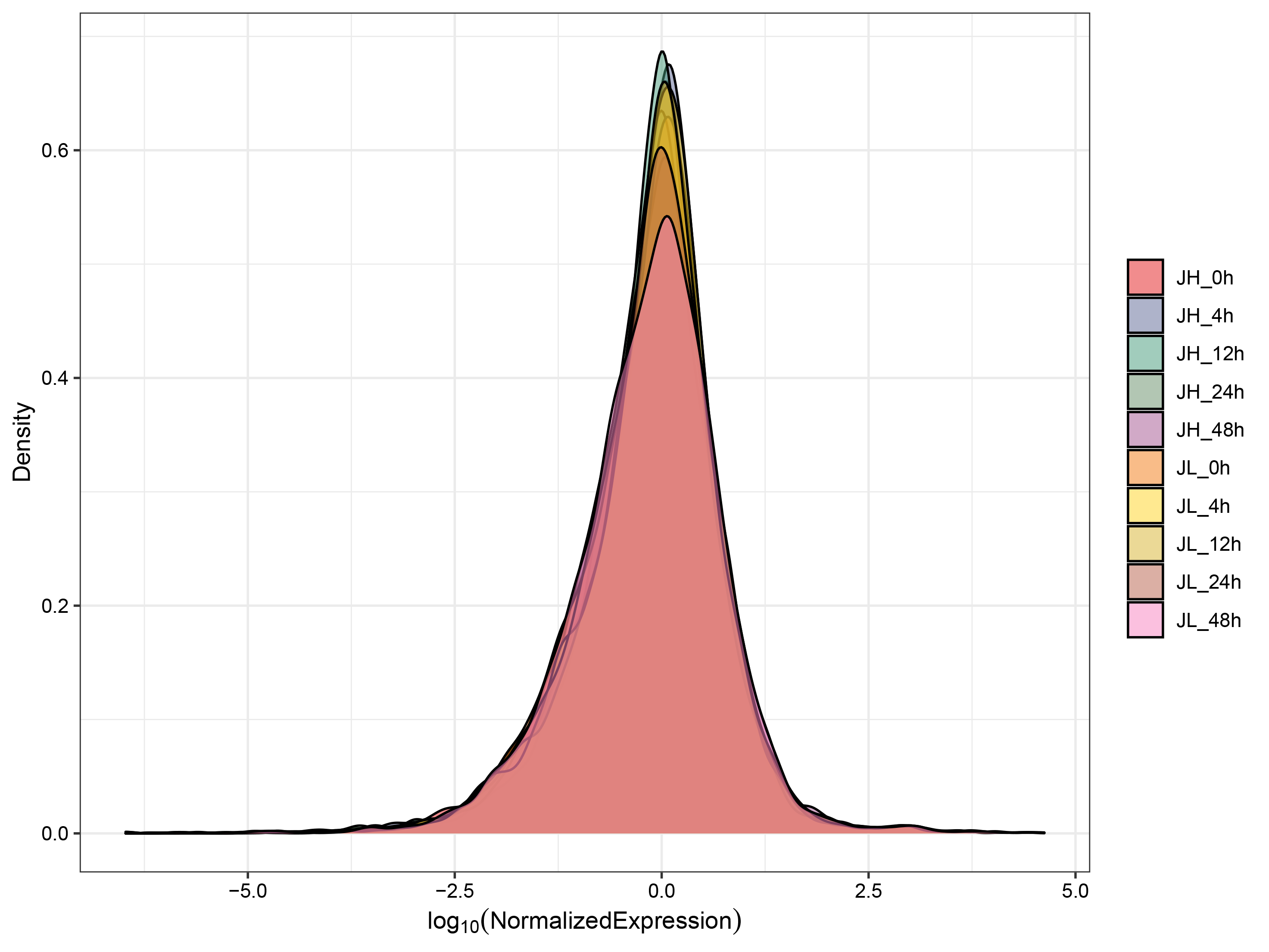

Supplement: Supplementary file 1 [file ijms-25-02310-s001.zip › Supplementary Figure S3.tif]

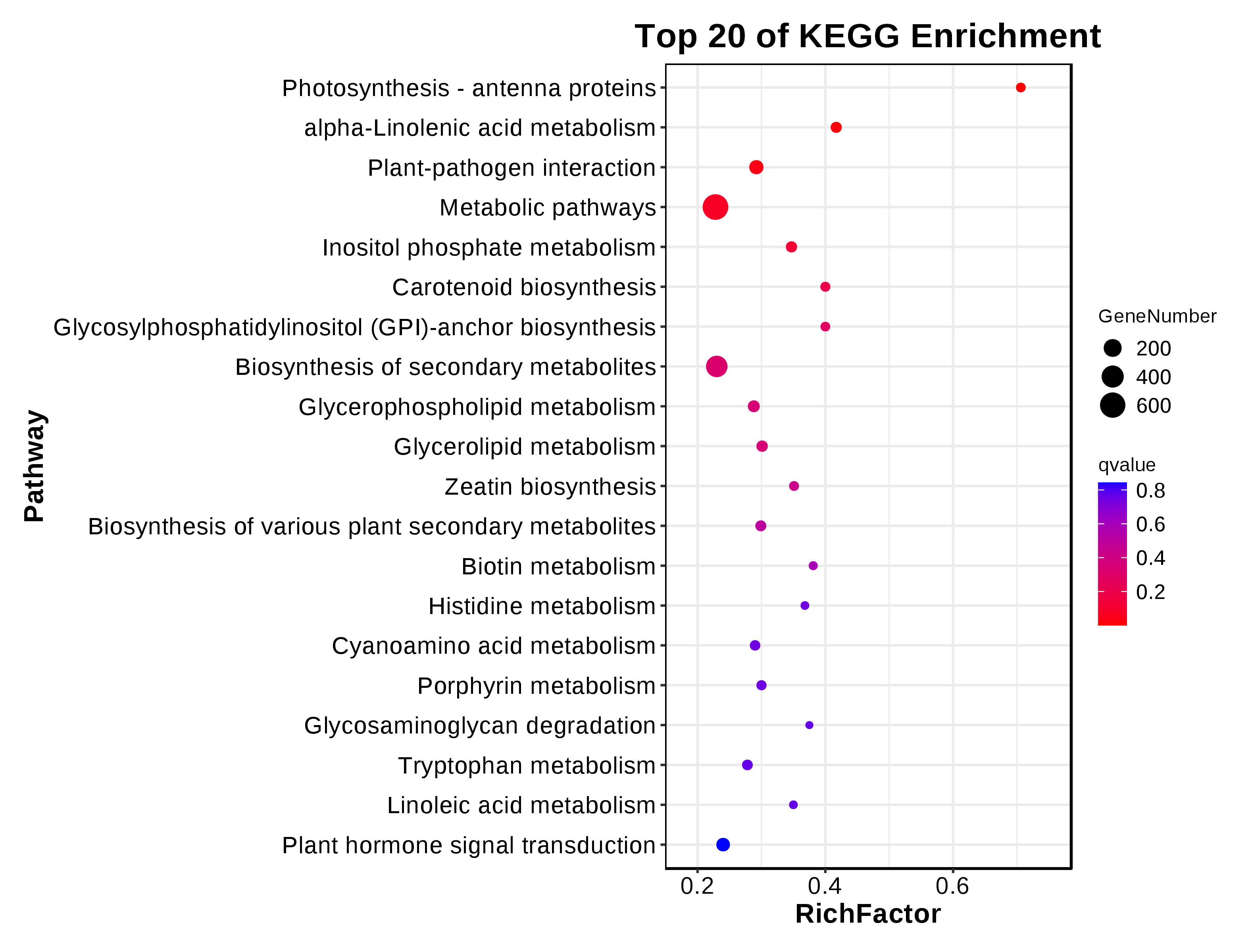

Supplement: Supplementary file 1 [file ijms-25-02310-s001.zip › Supplementary Figure S4.png]
